# Evaluation of Stepping Stones as a tool for changing knowledge, attitudes and behaviours associated with gender, relationships and HIV risk in Karnataka, India

**DOI:** 10.1186/1471-2458-11-496

**Published:** 2011-06-24

**Authors:** Janet E Bradley, Parinita Bhattacharjee, Banadakoppa M Ramesh, Meghna Girish, Arup K Das

**Affiliations:** 1 Centre for Global Public Health, Faculty of Medicine, University of Manitoba, 771 Mc Dermot Avenue, Medical Rehabilitation Building, Room R070, Winnipeg, Manitoba R3E 0T6, Canada; 2 Karnataka Health Promotion Trust, IT Park 5th floor, #1-4 Rajajinagar Industrial Area, Behind KSSIDC Admin Office, Rajajinagar, Bangalore 560 044, India

## Abstract

**Background:**

Stepping Stones training aims to help individuals explore sexual relationships and recognize gender inequalities, the structural drivers of the HIV epidemic, in order to understand risk behaviours and to seek solutions to factors that increase HIV vulnerability. Despite earlier studies suggesting the success of Stepping Stones, little data exist to show diffusion to trainees' social networks or the wider community.

**Methods:**

A mixed-methods evaluation of this approach was undertaken using in-depth interviews of trainees and friends, and polling booth surveys in 20 villages where Stepping Stones training took place and in another 20 villages with no Stepping Stones intervention.

**Results:**

The interview respondents and their friends reported significant changes in their relationships after training, and benefit from discussion of gender, sexuality, condom use and HIV vulnerability issues. However, though diffusion of this knowledge at the level of personal contacts was strong, the evaluation revealed that diffusion to the community level was limited.

**Conclusions:**

The qualitative part of this study reflects other studies in different settings, in that SS participants gained immensely from the training. Wider behaviour change is a challenging goal that many programmes fail to attain, with most interventions too limited in scope and intensity to produce larger community effects. This may have contributed to the fact that we observed few differences between interventions and non-intervention villages in this study. However, it is also possible that we had excessive expectations of individual change at the community level, and that it might have been more appropriate to have had broader community level rather than individual behavioural change indicators. We suggest that SS could be enhanced by efforts to better engage existing community opinion leaders, to empower and train participants as community change agents, and to support the development of village-level action plans that combat sexual stereotyping and risky behaviours that lead to unhealthy sexual relationships.

## Background

It is estimated that 2.5 million people are now living with HIV in India [1]. Though the national HIV prevalence of 0.36% is not suggestive of a generalized epidemic, HIV continues to affect large numbers of people. The Indian government acknowledges that some of the major challenges in dealing with the HIV epidemic are stigma, discrimination and gender inequality [2]. In order to prevent HIV infection, attention should be directed towards reducing risk behaviours, but also to the structural factors that affect vulnerability and risk [3]. Interventions at the individual level are thought to help people change, by providing knowledge or by attempting to alter beliefs, attitudes, perceived norms, motivation, and skills related to high risk activities. Interventions at the group and community levels attempt to modify social norms and to influence social networking, resources and opportunities and barriers to preventive practices in the community [4].

### Stepping Stones

Stepping Stones (SS) is a participatory training package [5] designed to address the prevention and spread of HIV and AIDS through promoting communication and relationship skills within households and communities. It aims to enable individuals and communities to find their own solutions to dealing with the reality of HIV/AIDS [6], to discover how to negotiate and cope through self-realization, learning, sharing and caring for those most affected. Theoretically, individual behaviour change is best achieved in the context of peer support and wider community changes, which includes rethinking negative social and cultural norms together [6].

During 2001-06, the Karnataka Health Promotion Trust (KHPT) undertook HIV/AIDS prevention activities in approximately 600 villages in Bagalkot district of Northern Karnataka, India. Stepping Stones was introduced by trained link workers as a key behaviour change tool in 202 of these villages. The project adapted the original SS manual to suit Indian conditions [7]. In most villages (median size 2100 people), 4 groups of people (married and unmarried men, married and unmarried women) were trained, with the training involving separate group trainings as well as occasional mixed group trainings and other meetings that were arranged for trainees to plan future joint community activities. Selected trainees were a mixture of people: some (around 15%) were invited to participate because link workers felt they were at high risk of HIV (sex workers were not included as there was a separate programme for them), some (around 60%) were invited because they were felt to be potential community change agents (such as health workers, teachers), and others (25%) volunteered, though their volunteering suggested they were interested in community development. In total, approximately 3400 women and 3400 men completed training in 202 villages. The drop-out rate was 15%, and this was highest among older men. KHPT expected that the trainees would not only benefit personally but would, as a group, take the ideas forward in their villages.

### Previous evaluations of Stepping Stones

In a summary of existing SS evaluations, it was noted that although SS had been widely used, much of the evidence of its success is based on short term reviews or on anecdotal data. However, all reports were overwhelmingly positive that SS is a valuable tool for individual change [6]. Most studies reported an improvement in communication, increase in knowledge and understanding of HIV/AIDS, and positive changes in behaviour, such as increased condom use, more respect for women, less domestic violence, better communication and more domestic co-operation [8-11]. More recently, a cluster randomized controlled trial in South Africa undertaken to measure the impact of SS on HIV and HSV-2 incidence and sexual behaviour showed that participation in the Stepping Stones programme was associated with a reduction in male and female herpes simplex type 2 (HSV-2) and intimate partner violence. The study showed, however, that Stepping Stones failed to significantly affect the incidence of HIV, and failed to positively affect reported female risk behaviours [12].

Although many studies have shown that SS training appears to benefit the participants, few data exist to show diffusion to the participants' social networks or the wider community, apart from studies in the Gambia and South Africa that suggested this might have happened [13,14]. As SS aims to bring about changes in community norms that support individual behaviour change, evidence for dissemination of SS learning at the community level is important.

### Stepping Stones evaluation in Karnataka, India

**Figure 1:**
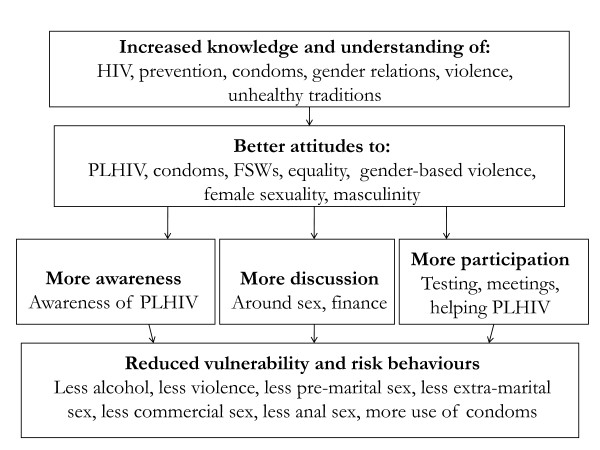
**Expected changes after SS intervention**.

In November 2007, we undertook a cross sectional evaluation of SS in Bagalkot District, 2-3 years after its implementation. We examined the impact of SS on personal knowledge, attitudes and behaviours (KAB) of SS participants and their close friends. At the community level, we examined KAB among general community members in a selection of 20 SS villages and 20 non-SS villages in the same district. Overall, the changes the programme implementers expected to see were increased knowledge of HIV/AIDS, more progressive behaviours at an intermediate level: more discussions in the home, more awareness of, and empathy with, people with HIV in their community, more participation in meetings and more HIV testing. Furthermore, they anticipated these changes would lead to reduction in risk behaviours such as violence against women, alcohol use, engaging in commercial sex, anal sex and sex without condoms (Figure 1). Our study had 3 specific aims based on the programme implementers' expected outcomes:

• To understand if and how the training had benefited the participants individually, by conducting individual interviews with past trainees, as well as polling booth surveys (PBS described below) that would show a significant difference in knowledge, attitudes and practices to the general population in their villages. The expectation was that the trainees would give positive feedback when interviewed individually and would also have more knowledge of STIs and HIV, more gender-sensitive, PLHIV-sensitive and egalitarian attitudes and practice safer sex than other people in their villages.

• To understand if and how the issues covered in the training had diffused to the participants' personal contacts by interviewing some close contacts of past trainees. The expectation was that the close contacts would also have positive views about SS and know some of the things learned by their trainee friends.

• To assess whether there had been any diffusion of ideas to the communities in which the training had taken place by comparing data from PBS among a sample of trainees, with people randomly selected in the general community in 20 villages where the trainees lived, and with another random sample of people in 20 villages where there had been no training. One purpose of the training in these villages was to have the trainee group work in their villages to spread the SS messages, organize other training and village activities around gender issues and HIV/AIDS. The expectation was that the SS ideas would have spread to the small communities in which they lived as demonstrated by better knowledge, attitudes and practices by people in their villages compared with people in villages where there had been no training.

## Methods

### Study design

We used a mixed methods approach, using quantitative and qualitative research methodologies to triangulate data on knowledge, attitudes and reported behaviours in SS participants, their immediate social networks and the wider community in Bagalkot district. We compared the SS community data with data from non-intervention communities. Two key techniques were used: 1) In-depth interviews were conducted with past trainees and their close contacts, and 2) polling booth surveys were conducted with past trainees, and general population members in their villages as well as in other villages with no such training. The sampling methods are described below for each research approach.

### Data collection methods

#### In-depth interviews (IDI)

Twenty villages where SS had been conducted were selected randomly from a total list of 200 villages where training had taken place between January 2004 and September 2006, i.e. 1-3 years before the survey. From each of these 20 SS villages, we selected one past trainee for a semi-structured, in-depth interview. Half were selected at random from a list prepared by KHPT staff of people whom they staff thought had benefited from training: the other half were selected at random from the larger list of trainees. Each of the selected SS trainees was also asked to identify a friend or relative for interview, someone with whom they shared a close relationship. They were not informed of the reason for the interviews. The interviews were conducted in the local vernacular by interviewers trained in semi-structured interviewing. Questions included their perspectives on the SS training, key knowledge gained and shared, perspectives on personal attitudinal and behavioural changes, and views on change attributable to SS, among their friends and in the community. All IDI transcripts were analyzed manually for repeated themes and ideas.

#### Polling Booth Surveys (PBS)

Polling Booth Surveys (PBS) have been used by KHPT and others to obtain information on delicate subjects such as sexual practices; the methodology has been reported elsewhere [15]. In summary, participants are interviewed in a group, though each person is behind a polling booth-type screen. The PBS sessions were conducted by trained PBS facilitators in the local vernacular in 40 villages: 20 where SS had been conducted in 2005, and 20 where no such training had been done. First we divided each village into 6 segments and randomly assigned each segment to one of the following 6 groups (unmarried women and unmarried men, younger married women and men, older married women and men). In each segment, one house was selected at random as a "starter" house. In this home, we listed all members of the household. If any person satisfied the criteria for inclusion, we requested their participation (if there was more than one such person in the house, we selected one at random). Then working to the left, we followed the same procedure in each house until we had invited 12 people in the appropriate group, giving an approximate total invited sample of 1440 respondents in the 20 SS villages and 1440 in the 20 non-SS villages. We were able to poll 1196 respondents in SS villages (83% response) and 1297 (90% response) in other villages, with a combined sample of 2493 (87% response). In addition, as many SS trainees as possible from the 20 Stepping Stones villages, were convenience-sampled to participate in a separate PBS session. In all 414 former trainees were sampled. Questions were a mixture of ones used in previous KHPT PBS and some were taken from the GEM Scale [16]. The questions focused on knowledge, attitude and behaviour and involved yes/no or don't know/not applicable responses. The knowledge and attitude questions were the same for all groups, but the behaviour questions differed according to what was appropriate for the specific group. All PBS data were entered into Excel spreadsheets and differences between groups were calculated using a chi-squared test. Preliminary analysis found that the general population samples were very similar; however the SS group profile was different to that of the general population samples (p < 0.01, Table 1), so the data from the SS trainee group were adjusted directly, using the stratum-specific denominator of the SS general population group as the standard population. Differences between groups were then calculated in STATA version 10 (STATA Corporation, USA) and *p* values calculated using a z-test (test of equality between proportions).

**Table 1:** PBS respondent profiles

Respondents	20 SS villages SS trainees N = 414	20 SS villages General population N = 1196	20 Non SS villages General population N = 1277
Married women 15-29	11%	18%	17%
Married women 30-49	24%	17%	16%
Married men 15-29	20%	16%	17%
Married men 30-49	12%	17%	17%
Unmarried women 15-24	10%	17%	17%
Unmarried men 15-24	23%	16%	17%

χ^2 ^(10) = 46.3132 p < 0.01

### Ethical considerations

All efforts were made to ensure the privacy of the survey respondents. No names were used in any of the recorded data, and verbal, witnessed informed consent was obtained from all participants. The study was approved by the Ethics Review Board of St John's Medical College, Bangalore.

## Results

### In-depth interviews with SS trainees and friends

Of 20 people approached, 19 in-depth interviews were completed with Stepping Stones (SS) participants (10 males and 9 females), all from different villages. The other audio tape was inaudible and was discarded. At the time of training in 2005, half these participants were married and half unmarried, but at the time of the interviews in November 2007, 7/10 men were married as were 5/9 women. The mean age of the male respondents was 24 and of the women was 28.4 years.

Twenty friends (SSF) were also interviewed but again one audio tape was inaudible and was discarded, so we completed 19 interviews (SSF) (10 males and 9 females) with people from different villages. All were friends of the SS participants, though one was also a cousin. Nobody brought along close relatives or anyone of the opposite sex. Of the 10 SSF males, 3 were married; of the 9 SSF women, 6 were married. The mean age of the SSF male respondents was 23.5 and of the SSF women was 26.4 years.

Most SS and SSF respondents had completed high levels of formal education. Despite this, most were engaged in manual jobs or were unemployed. Of the SS participants, four of the men classified themselves as farmers, one as a student and the others had manual jobs such as carpenter, shop assistant, cable operator. Four of the SS women classified themselves as farmers, 3 as unemployed, one as a tailor and one as an *anganawadi *(village health worker). Of the SSF male respondents, most were in manual occupations, though one was a teacher, one a student and one a driver. Of the SSF women, 4 identified as farmers, 3 as unemployed, one as a tailor and one as a cook.

All 19 participants, irrespective of how they were selected, reported being very happy with the training: they commented that it was extremely relevant to their lives, had opened their eyes to key issues, made them think about their relationships and had helped them to change. Most comments reflected that SS training had helped growth and maturity, preparing them for a happy family life. One young man commented "SS is not just useful-it shapes our lives. Just as we can purify dirty water, so has SS made my life clear and sparkling."

One young woman's comments epitomized the general consensus about the training:

"I feel my capacity has been built to deal with any situation and face any problem in life. I am getting married and I feel this training will be with me forever. I am prepared for entering married life and a sexual relationship with an emphasis on safety. I am confident that I can speak to my husband about my feelings and about sex, what I like and dislike, and about protection through condom use. I knew nothing about the joys and sorrows of sex before."

Surprisingly respondents could recall much of what had been taught 2-3 years earlier and often could remember the names of the specific sessions. Most said that they particularly enjoyed those sessions that focused on loving relationships. Although discussions about sex are rather taboo in Indian society, especially for unmarried boys and girls, every respondent affirmed that sex should be a key area of discussion in SS training. Seven participants said that at first they felt uncomfortable or embarrassed, but all agreed that they had learnt a lot, and that it was very important for their health and life: "life can get very badly affected because of secrecy and shyness. Discussions about these things lead people to seek treatment, support HIV prevention efforts and leads to better health for all". Married women felt that the discussions of sex gave them the information they needed to confront spouses: "I told my husband about sexual relationships and the HIV infection that spreads through sex. A wife has to talk to her husband to protect herself...I took condoms from the clinic and he used them."

All respondents, irrespective of how they were selected, were vocal when asked about gender relations in the community. Many mentioned that there was a problem in a society that treated women as inferior, where girls were not encouraged to go to school or have careers, where men and women were prevented from talking openly together, where early marriage and marriage of nieces or first cousins was the norm, and most importantly, when men perpetrated violence against women. The males themselves mentioned that they used to think that girls who talked to boys were "loose" or available for sex, but that SS had changed their ideas. The SS respondents reported that the training had made them personally reflect on what needed to be done to improve equality. Many reported that gender relations in their villages had improved after the SS training, starting by them modelling behaviours in their own home and with their peers.

All respondents also reported gaining new information on HIV and other STIs, how to manage healthy relationships and once they understood about HIV, the need to reduce stigma against people with HIV/AIDS (PLHIV). The men mostly reported changes in personal behaviours, especially reduction in alcohol use, reduction in multi-partner sex, increase in condom use and increased respectfulness towards women. The women mostly reported an increase in courage and confidence to face issues. Their testimonies reveal a strong sense of pride: "I have increased self-confidence...in my family, my parents are happy that their daughter is a complete young woman, that even in marriage she knows how to have a fulfilled life. Now I tell my parents what my dreams are, how I have confidence to face problems when they crop up".

All respondents, irrespective of how they were selected, commented that they had shared their new ideas with friends and relatives, despite not being formally encouraged to do this during the training. They also mentioned that there was little support from the facilitators to stay together as a group and take on concrete community activities after training. Despite this, they noted such changes in the community as: more discussions, more knowledge about sex and HIV, and less fear of HIV, more self-help groups who were generally improving the village; more respect for women, improved gender relations, more girls in school; more testing and treatment for diseases like STIs; a reduction in smoking, drinking, risky sex and violence against women; an improvement in attitudes to *devadasi *(traditional caste-based sex workers); general increased levels of responsibility and use of condoms.

SS trainees' associates were interviewed to triangulate some of the responses given by the SS respondents. All 19 respondents remembered that their friends went for this training and all remembered being told about it. Most males specifically mentioned that their friend had told them about HIV/AIDS, how to use condoms and how to reduce risk. They had also learnt from them about caring for positive people (PLHIV) and about being tested themselves. The respondents noted also that their SS friend had discussed with them appropriate behaviour with, and respect for, girls. Among the female associates, information sharing appeared to be slightly different, with recollections much broader than just reduction of risk behaviours and HIV. They were more likely to mention issues such as how to communicate with people, how to develop healthy family relationships, trust and confidentiality, how to care for PLHIV, how to be self-confident and take on issues of importance, how to challenge unhealthy traditions, how to convince men to behave more responsibly in the areas of drinking and womanizing and how to love and keep your man at home.

### Polling booth results

Table 1 shows the profiles of the 2,887 people who participated in the PBS. The profiles of the general populations were very similar. However, there were a greater number of older married women, younger married and unmarried males among the SS trainees than in the general population samples. Adjusted data for the SS group are presented.

All participants answered every question by putting a token in one of three boxes, and we present the "yes' answers (agree answers in the case of the attitude questions). Two people in the SS sample left before answering the attitude section of the PBS. Tables 2, 3 and 4 show the different responses in the three study populations. Where the differences were statistically significant from the population in the previous column (for example, comparing SS trainees with people in their villages, or comparing people in SS villages with people in non-SS villages), asterisks show statistical significance; (*p*< 0.05, one asterisk) or (*p*< 0.01, two asterisks).

**Table 2:** HIV knowledge (% respondents responding "yes" to the statement)

	SS trainees N = 414	SS villages General population N = 1196	Non-SS villages General population N = 1297
Have you have heard of HIV?	92.8	75.0 (0.00)**	72.5 (0.16)
Have you ever received any information about HIV?	79.7	57.9 (0.00)**	56.1 (0.36)
Can HIV be transmitted by mosquito?	29.8	28.8 (0.70)	44.3 (0.00)**
Can HIV be transmitted by sex?	76.1	58.0 (0.00)**	53.6 (0.27)*
Can you tell by looking who has HIV?	26.8	23.4 (0.16)	37.6 (0.00)**
Do you think condoms protect against HIV?	72.4	53.8 (0.00)**	69.5 (0.00)**
Do you think anal sex is safe and a way to avoid HIV?	25.4	21.5 (0.10)	34.6 (0.00)**
Do you know where to get condoms?	76.4	59.4 (0.00)**	56.9 (0.21)
Have you ever seen a demonstration on how to put on a condom?	61.2	39.7 (0.00)**	34.1 (0.00)**

*p *< 0.05*

*p *< 0.01**

**Table 3:** Attitudes (% respondents agreeing with the statement)

	SS trainees N = 412	SS villages General population N = 1196	Non-SS villages General population N = 1297
**Attitudes to HIV and PLHIV**			
I would care for a PLHIV relative at home	77.2	64.9 (0.00)**	60.2 (0.02)*
I would not buy vegetables from a person with HIV	39.6	47.5 (0.00)**	46.5 (0.62)
HIV child should not be allowed in school	30.1	40.4 (0.00)**	40.9 (0.80)
If a woman has HIV, it is a reflection of moral character	44.6	46.8 (0.44)	46.4 (0.84)
Families with HIV should be left alone	33.7	37.8 (0.14)	40.9 (0.11)
			
**Gender and sexuality**			
It's OK for women to suggest condom use	72.8	58.0 (0.00)**	54.8 (0.11)
Girls with too much education are not good wives	31.2	39.5 (0.00)**	44.3 (0.02)*
Girls should be married as soon as possible	45.8	52.5 (0.02)*	51.6 (0.65)
Women should feel free to show husbands if they want sex	59.7	53.6 (0.03)*	51.4 (0.27)
Women should be blamed for spreading AIDS	29.3	32.3 (0.26)	38.7 (0.00)**
Men who cook are not real men	76.5	72.6 (0.12)	72.0 (0.74)
			
**Caring and sharing**			
You don't talk about sex, you just do it	42.7	50.7 (0.00)**	46.4 (0.03)*
It's a woman's responsibility to avoid pregnancy	38.2	45.7 (0.00)**	43.5 (0.27)
Man should have the final word about decisions in the home	52.9	64.0 (0.00)**	63.4 (0.76)
An ideal husband controls his wife	71.9	75.6 (0.14)	72.5 (0.08)
Women should give their earnings to the husband	58.4	64.9 (0.02)*	67.2 (0.23)
			
**Gender based violence**			
Men cannot control their sexual urges	49.4	44.8 (0.11)	51.2 (0.00)**
It's OK for a man to force his wife to have sex	39.8	42.0 (0.43)	50.3 (0.00)**
There are times when a woman should be beaten	53.8	57.4 (0.20)	60.4 (0.13)
Raped women are usually at fault	31.6	30.4 (0.65)	34. 1 (0.05)

*p *< 0.05*

*p *< 0.01**

**Table 4:** Behaviours (% respondents responding "yes" to the statement)

	SS trainees N = 414	SS villages General population N = 1196	Non-SS villages General population N = 1297
**Openness to new ideas**			
Have you discussed sex with spouse in last 6 months	46.1	32.9 (0.00)**	35.6 (0.16)
Do you discuss finances with your spouse?	60.1	45.0 (0.00)**	44.1 (0.65)
Have you been to a meeting about HIV in last 6 months?	61.1	40.6 (0.00)**	40.7 (0.96)
Do you know any people with HIV/AIDS?	51.0	35.7 (0.00)**	36.0 (0.88)
Of those who know a PLHIV, have you ever helped a PLHIV?	60.8	58.1 (0.34)	45.0 (0.00)**
Would you be willing to be tested for HIV?	57.4	46.7 (0.00)**	44.1 (0.19)
Have you been tested for HIV?	24.0	16.9 (0.00)**	16.4 (0.74)
			
**Alcohol and forced sex**			
Do you use alcohol? (Men only)	38.3	42.8 (0.11)	48.9 (0.00)**
Does your husband consume alcohol (Married women only)?	28.6	30.7 (0.42)	36.5 (0.00)**
Have you forced any woman to have sex in last 6 months? (Men only)	9.0	5.0 (0.00)**	7.2 (0.02)*
			
**Risky behaviours**			
Have you had more than 1 partner in last 6 months?	8.1	9.8 (0.31)	14.9 (0.00)**
Was a condom used every time had ex-marital sex? (Married people only)	66.6	34.3 (0.00)**	45.7 (0.00)**
If more than 1 partner, was a condom used every time? (Unmarried people only)	83.6	51.7 (0.00)**	55.4 (0.06)
Have you been to a sex worker in the last 6 months? (Men only)	4.9	6.0 (0.41)	10.8 (0.00)**
On the last time you had sex with a sex worker, was a condom used? (Men only)	59.1	43.2 (0.00)**	48.7 (0.00)**
Did you have anal sex with a man in last 6 months? (Men only)	5.8	2.2 (0.00)**	3.1 (0.16)
Did you use a condom the last time you had anal sex with a man? (Men only)	44.0	29.0 (0.00)**	35.4 (0.00)**

*p *< 0.05*

*p *< 0.01**

#### Knowledge of HIV and transmission modes

Overall, the SS trainees had more knowledge than the general population and in turn the SS villages' general population was more knowledgeable than people in the non-SS villages (Table 2). The SS trainees were more likely to have heard of HIV/AIDS than the general population (92.8% vs.75%, *p *< 0.01), more likely to have had specific information (79.7% vs. 57.9%, *p *< 0.01), and more likely to know that HIV can be transmitted sexually (76.1% vs. 58.0%, *p *< 0.01). SS trainees were much more likely to know that condoms can protect against HIV than the general population (72.4% vs. 53.8%, *p *< 0.01), to know where to get a condom (76.4% vs. 59.4%, *p *< 0.01) and more likely to have seen a condom demonstration (61.2% vs.39.7%, *p *< 0.01). On many of these indicators, the people living in SS villages were much more knowledgeable than people in non-SS villages.

#### Attitudes to PLHIV

Five questions were asked to all respondents about their attitudes to people living with HIV/AIDS (Table 3). On three of the indicators, whether they would care for a PLHIV in the home (77.2% vs. 64.9%,* p* < 0.01), whether they would buy vegetables from a PLHIV (60.4% vs.52.5%, *p* < 0.01) and whether a child with HIV should be allowed in school (69.9% vs. 59.6%, *p* < 0.01), the SS trainees scored higher than the general population in their villages and differences were all were statistically significant.

Very little difference was seen between the general populations of the two types of villages: only the different answers to the question about caring for a PLHIV were statistically significant (64.9% vs. 60.2%, *p *= 0.02). Overall, more than one third of the respondents had negative attitudes to PLHIV. Almost half the respondents in all groups, including those who had been trained in SS, thought that being HIV positive was a reflection of moral character.

#### Gender and sexuality

On indicators related to female roles, the SS trainees again showed more gender equitable attitudes than the general population (Table 3). The general population respondents were more likely than SS respondents to agree with the statement that girls with too much education do not make good wives (39.5% vs. 31.2% respectively, *p *< 0.01), more likely to agree that women should be blamed for spreading HIV (32.3% vs. 29.3%, not significant (ns), and less likely to think that it is OK for women to suggest condom use with their husbands (58.0% vs.72.8%, *p *< 0.01). However, even among the SS trainees, many had deeply ingrained socially-sanctioned attitudes to marriage, to whether women are responsible for HIV, and about whether women should make sexual advances to their spouses.

The general population of SS villages had slightly more progressive views than the general population in non-SS villages in some areas, for example 39.5% of those in SS villages believed that girls with too much education do not make good wives, compared to 44.3% of those in non-SS villages (*p *= 0.02) and fewer believed that women should be blamed for spreading HIV (32.3% vs. 38.7%, *p *< 0.01). In other areas, there seemed to be little difference in attitudes between those villages with an SS intervention and those without. The idea of men cooking appeared to be an anathema in all groups.

#### Attitudes to sharing, caring and responsibility

After training, we expected that SS trainees and people in their villages would show more gender-progressive attitudes, with respect to sharing responsibility and decision-making in the home. In general, people were fairly progressive in their ideas about sharing responsibility for contraception, for example, but on other issues, SS participants were seen to hold fairly regressive views. However, again, on all indicators, the SS trainees fared better than others; they were much less likely to agree with statements, such as "you don't talk about sex, just do it" (42.7% vs. 50.7%, *p *< 0.01), that it is a woman's responsibility to avoid pregnancy (38.2% vs. 45.7%, *p *< 0.01), that men should always have the final word about decisions in the home (52.9% vs. 64.0%, *p *< 0.01), that an ideal husband controls his wife (71.9% vs. 75.6%, ns), and that women should give their earnings to their husbands (58.4% vs. 64.9%, *p *= 0.02). However, again, the views of people in SS villages were not significantly different from those of respondents in non-SS villages.

#### Attitudes to gender based violence and forced sex

Gender, violence and forced sex are key themes discussed in SS training and our hypothesis was that the SS trainees therefore would be adamantly against such acts. However, the PBS data showed that conventional norms are deep-seated in all groups surveyed, with a large proportion of all respondents agreeing that men could not control their sexual urges, that it is fine for a man to force his wife to have sex, and that there are times when a wife deserves to be beaten. One third of all SS respondents also believed that raped women are usually at fault. On all these issues, the SS trainees did not fare much better than other respondents in their villages although both were more enlightened than respondents in non-SS villages. Respondents in non-SS villages were more likely than those in SS villages to agree that men are unable to control sexual urges (51.2% vs. 44.8%, *p *< 0.01) and that it is permissible for a man to force his wife to have sex (50.3% vs. 42.0%, *p *< 0.01).

#### Behaviours: openness to new ideas

The programme implementers' expectation was that before changing major risk behaviours, people exposed to SS ideas, their friends and in turn their communities, would start to discuss sensitive issues in the home, would become more aware of HIV in their communities, be more pro-active about assisting PLHIV, and would consider HIV testing. In all these areas the SS trainees reported considerably more positive behaviours than the general population in the SS villages (Table 4). For example, they were much more likely to report recently discussing sex with their spouse (46.1% vs. 32.9%, *p *< 0.01), and discussing financial issues (60.1% vs. 45.0%, *p *< 0.01). They were also much more likely to say that they had been to a meeting about HIV (61.1% vs.40.6%, *p *< 0.01), that they knew someone with HIV/AIDS (51.0% vs. 35.7%, *p *< 0.01) and that they had personally provided help to a PLHIV (60.8% vs. 58.1%, ns). Furthermore, they were more likely to consider HIV testing (57.4% vs. 46.7%, *p *< 0.01) and to actually have been tested (24.0% vs.16.9%, *p *< 0.01).

Comparing the general population in SS and non-SS villages, there were no differences observed, except on the indicator relating to helping a PLHIV (58.1% vs. 45.0%, *p *< 0.01).

#### Behaviour: alcohol use and forced sex

Men were asked about their use of alcohol and perpetration of forced sex. Reported alcohol use was lower among SS male trainees than men in the general population in their villages (38.3% vs. 42.8%, ns), and this in turn was lower than in non-SS villages (48.9%, *p *< 0.05). We also asked men if they had *ever *forced a woman, including their wife, to have sex. Overall, 15% of men admitted this; 13% of young married men, 18% of older married men and 10% of unmarried men (data not shown). Despite training, forced sex (in the last 6 months) was reported significantly more in the trainee group than in the general population in their villages (9.0% vs. 5.0%, *p* < 0.01), but the rate in the SS villages was significantly less than in other villages (5.0% vs. 7.2%,* p* = 0.02).

#### Behaviour: risky sex

This series of indicators looked at multiple sex partners, sex with FSWs, anal sex, and use of condoms in these situations. Overall, approximately 12% of married women and 30% of married men reported ever having extra-marital sex. Among unmarried respondents, 5% of women and 15% of men reported having more than one partner in the previous six months. Overall, 15% of men reported that they had ever had sex with a sex worker; older married men were more likely to have ever done this (21%) than younger married men (15%) and unmarried men (10%).

Comparing the different groups of respondents, the results are varied. First, there was no difference between SS trainees and people in SS villages with respect to having multiple partners in the last 6 months (8.1% vs. 9.8%, ns), but the SS trainees were slightly less likely to report having had sex with an FSW in the previous 6 months (4.9% vs. 6.0%, ns). On the other hand, 5.8% of SS male trainees reported anal sex with a man in the previous 6 months, compared with only 2.2% of SS village general male population (*p *< 0.01). Although the number of men reporting engaging in risk behaviours was small in all three groups, the use of condoms in these encounters was overall less than half. Even some of the men trained in SS reported risky behaviour and lack of protection, although they were more likely to use condoms than men in the SS villages. Use of condoms for SS married people who had sex outside marriage was 66.6% compared with 34.3% of married people in the general population (*p *< 0.01). SS men were also more likely to report condom use when having sex with an FSW (59.1% vs. 43.2%, *p* < 0.00), or with another man (44.0% vs. 29.0%, *p* < 0.01).

Paradoxically, when comparing the SS villages general population with the non-SS villages general population, the former were less likely to report risk behaviours, but also *less *likely than men in non-SS villages to report condom use in these situations.

## Discussion

This study suffered from a few limitations. First, there is always a possibility of social desirability bias where people will state the social norm as the answer to a gender attitude questions even if their own views somewhat differ. However, the testimonies of the SS trainees, and also of their friends, were so strong and convincing that they did not seem designed only to please. Not only were all respondents able to passionately articulate their personal journeys, but also they had vivid recall of many of the training sessions that had taken place 2-3 years before. Second, the study suffered from lack of baseline data with which to be able to show change over time and to assure ourselves that the villages selected were comparable; we had limited data on the level of risk that existed in the villages before the training, though latterly we looked at the total number of sex workers and found that there were slightly more in the SS villages, averaging 1:153 population compared to 1:181 in the non-SS villages. Third, because the PBS samples were selected differently, that is the general population samples were selected using a stratified random cluster design and the SS trainees were convenience-sampled, the groups had slightly different profiles. The general population polling booth selection was also done without collating information on the size of the overall sampling frame, so cluster weights could not be assigned; furthermore, all PBS data were pooled, so it was not possible to perform any regression analyses that would control for population differences. However we did correct for these population differences using direct standardization. We also had no clear picture of the quality and completeness of the training sessions or of the attendance rates, making interpretation of some of the data, for example why so few people saw a condom demonstration, speculative. In a study like this, there is always an issue of being able to attribute the findings to the intervention in question. However, we feel that the large sample sizes, both the number of villages and the number of respondents, and the consistency of the results, can give us some assurance that the differences, especially between the trainees and the general populations were associated with Stepping Stones.

Stepping Stones has been described as an "individually-focused" intervention [14] and it was clear from the qualitative part of the study that the individuals trained SS loved the training and felt immense benefit. The men generally reported that they had become more responsible and more agreeable, and that they had reduced their risk-taking behaviours, such as drinking alcohol and visiting sex workers. The women on the other hand, were more likely to report that SS training had led to their empowerment and growing ability to discuss issues, to demand change at home and to improve their marriages, reflecting findings of other SS evaluations [6-14,17]. It is impressive that participants, and even their friends, could talk about the names and content of many of the individual training sessions several years after the training. These data were supported in part by the PBS data that found that SS trainees were more knowledgeable, had better attitudes and less risky behaviour than others in their communities. However, even among the trainees, there appeared still to be several misconceptions about HIV transmission. Our feeling is that as with any good quality small-group interactive training [18-21] participants liked the sessions and understood some of the general tenets of respectful relationships and healthy sexuality; however, this was to some extent at the expense of clear objective factual information. Also, despite the focus on gender issues, poor attitudes to women and violence, and to female sexuality and masculinity persisted, even where behaviours had improved. Jewkes suggests that while female participants in the South African study showed greater assertiveness and agency, they failed to challenge existing gender norms of conservative femininities [14]. In India, a deeply conservative society, it may be that we have exaggerated expectations of Stepping Stones as a transformative agent to tackle structural drivers of the epidemic, since gender roles and violence are so deep-rooted; for example, two-thirds of women and men in the National Family Health Survey in Karnataka in 2005 thought that it was justifiable for a man to beat his wife in some circumstances [22]. However, there were reported changes in behaviour, notwithstanding the absence of fundamental changes in gender perspectives.

The implementers' expectation was that the training would lead to significant changes at the community level, yet this was not observed. Wider behaviour and health change are challenging goals that many programmes fail to attain [23,24] with most interventions too limited in scope and intensity to produce larger community effects unless the communities are small and homogeneous, such as MSM populations [19,20,23]. A successful and sustainable community-level programme that can produce change in the social environment, must have multiple interventions that take account of cultural and environmental influences at the individual, community and policy levels, with emphasis on participation, mobilization and ownership by existing bodies such as women's groups and village health committees [23,24]. Apart from initial and final community meetings in each village, this project did not appear to follow these basic tenets; trainees appeared to have no formal "contract" with the existing village social structures, respondents told us they rarely met after training and that there was limited support for them to take action as a group; most people reported that after the training they felt let down or abandoned. In some cases, funding ended and the link workers who had conducted the training no longer came to the villages. In some villages, trainees organized themselves and had enough initiative to take on a specific issue, but in other villages, it seems that after the training, little happened. Even if there had been such community involvement, the training itself only has a couple of hours devoted to action planning and no specific training on strategies for working in the community arena.

A key issue, for which there exists little guidance, is what critical mass is needed to effectively take forward what, in this context, are radical new ideas. Jewkes suggests workshops should be offered on a wider scale within each community [14], but how wide? And what types of trainees are best equipped to do this? Indeed, the marginalized high risk nature of some of the Indian SS participants may in fact exempt their involvement in activities in more mainstream society. Another question is whether we had excessive expectations of individual change at the community level and that it might have been more appropriate to have broader community level rather than individual behavioural change indicators [25]. Indeed it has been argued that broader and smaller community level effects are indeed meaningful, as a modest reduction in risk can have a large public health impact [26].

## Conclusions

In conclusion, our qualitative study reflects other studies in different settings, in that SS participants gained immensely from the training. This study goes a further by confirming that intimate contacts of trainees also felt the benefits. However, despite the implementers' expectation that the intervention would be sufficient for change at the village level, this did not in fact happen. In a country of over one billion people, many of whom have very conservative gender-stereotypical attitudes that can contribute to the structural drivers of an HIV epidemic, an approach like Stepping Stones can play a role in fundamental changes in personal attitudes; however given the size of the population, this approach is untenable if short-term, and unsupported by additional mechanisms to promote ownership of cultural change at the community level. We suggest that SS can be enhanced by efforts to better engage existing community opinion leaders, to empower and train participants as community change agents, and to support the development of village-level action plans that combat sexual stereotyping and risky behaviours that lead to unhealthy sexual relationships.

## Competing interests

The authors declare that they have no financial competing interest. PB and MG developed the Indian version of the Stepping Stones training and managed training sessions, but declare no bias in implementing this study and no financial gain from the manual.

## Authors' contributions

JB developed the study, managed all aspects of the study, supervised the data analysis and wrote the manuscript. PB developed the original idea for the study, was involved in data interpretation and worked on the manuscript. MG undertook all the qualitative fieldwork, trained interviewers and managed the data analysis. BMR and AKR supervised the data analysis and made comments on the manuscript. All authors read and approved the final manuscript.

## Pre-publication history

The pre-publication history for this paper can be accessed here:

http://www.biomedcentral.com/1471-2458/11/496/prepub
